# Oil sludge washing with surfactants and co-solvents: oil recovery from different types of oil sludges

**DOI:** 10.1007/s11356-020-10591-9

**Published:** 2020-09-25

**Authors:** Diego Ramirez, Liz J. Shaw, Chris D. Collins

**Affiliations:** Department of Geography and Environmental Science, University of Reading, Reading, RG6 6DW UK

**Keywords:** Oil sludge washing (OSW), Surfactants, Co-solvents, Oil recovery rate (ORR), Hansen solubility parameter (HSP), Cyclohexane, Rhamnolipid

## Abstract

Different physicochemical and biological treatments have been used to treat oil sludges, and oil recovery techniques are preferred such as oil sludge washing (OSW) with surfactants and co-solvents. Toluene is commonly used as co-solvent, but it is non-benign to the environment. This study tested alternative co-solvents (*n*-pentane, *n*-hexane, cyclohexane, and isooctane) at 1:1 and 2:1 C/OS (co-solvent to oil sludge ratio). Also, this study evaluated the effect on the oil recovery rate (ORR) of three main parameters in the washing: type, concentration, and application ratio (S/OS) of surfactants to oil sludges. To date, no study has assessed these parameters in the washing of oil sludges from different sources. Four types of oil sludges and five surfactants (Triton X-100 and X-114, Tween 80, sodium dodecyl sulphate (SDS), and rhamnolipid) were used. The results showed that cyclohexane had high ORR and could be used instead of toluene because it is more benign to the environment. The S/OS ratio had a high effect on the ORR and depended on the type of oil sludge. Rhamnolipid, Triton X-100, and Triton X-114 had the highest oil recovery rates (40 – 70%). In addition, it was found that the surfactant concentration had no effect on the ORR. Consequently, the addition of surfactant was not significantly different compared to the washing with no surfactants, except for one sludge. The use of the surfactant in the washing solution can help in the selective extraction of specific oil hydrocarbon fractions in the recovered oil to assess its potential reuse as fuel. Further recommendations were given to improve the OSW process.

**Electronic supplementary material:**

The online version of this article (10.1007/s11356-020-10591-9) contains supplementary material, which is available to authorized users.

## Introduction

Oil sludges are hazardous wastes from the oil industry which are mainly comprised of crude oil, water, sediments, and metals (Hu et al. 2013). The amount of oil sludges is about 160 million tonnes per year (ANP 2010) with more than one billion tonnes accumulated worldwide (Mirghaffari 2017). Treatment of oil sludges focuses on physicochemical and biological remediation methods. It has been established that the oil sludge treatment should follow the reduction, reuse, and recycle (3R) policies mentioned in the present waste management procedures (Sakai et al. 2011; European Parliament 2008). Therefore, oil sludge washing (OSW) with surfactants has been used to recover the oil (Liang et al. 2017; Duan et al. 2018; Liu et al. 2018a; Chen et al. 2019) and sometimes co-solvents are added to help with the oil extraction process (Zheng et al. 2012). Hu et al. (2020) mentioned that there has been recently increasing oil recovery-related research to extract valuable energy and to reduce potentially harmful petroleum hydrocarbons and volume of oil sludge to dispose of.

The use of surfactants in the OSW process allows the demulsification of the water-in-oil type (W/O) emulsions from the oil sludges by decreasing the interfacial tension due to their amphiphilic state. The emulsions can then break due to the continuous agitation during the washing (Rosen and Kunjappu 2012). Ramirez and Collins (2018) reported that the surfactant type, concentration and application ratio to oil sludge (S/OS) are relevant in the OSW process because these parameters can influence the oil recovery. In that study, it was reported a maximum oil recovery rate (ORR) from an oil-water separator sludge at low S/OS ratios and surfactant concentrations. Briefly, the study established that the S/OS ratio had the strongest effect in maximizing the recovery. The surfactants with the best oil recovery rates were Triton X-100 (32% ± 5), rhamnolipid (29% ± 8), and Triton X-114 (30% ± 7), and the overall optimal surfactant concentration was 2CMC (critical micelle concentration). Sodium dodecyl sulphate (SDS) and Tween 80 had lower recoveries (less than 15%). Toluene was used as a co-solvent in the study at a 1:1 co-solvent to oil sludge ratio (C/OS).

Co-solvents are also added in the OSW to assist in the extraction of the oil (Schramm 2000) that has been previously demulsified by the surfactants. The rationale behind the use of a co-solvent in the oil recovery is the selective extraction of all oil components from sludge, and therefore, the miscibility of the solvent with the oil is determinant in the success of the oil extraction (Rincón et al. 2005; Hu et al. 2017). In addition, the solvent can repel chemical additives used in the oil industry and the dispersed particles from the oil/solvent solution. Then, the sedimentation of unwanted particles by gravitation can be facilitated (Rincón et al. 2005). Toluene is commonly used in oil recovery studies, but it is not benign to the environment and human health (Fishbein 1985; Young 2007b; Wacławek et al. 2016). Therefore, it is necessary to test alternative organic co-solvents that are less harmful to the environment. To our knowledge, no studies have analysed the effect of these co-solvents in the oil recovery from oil sludges.

The co-solvents chosen for this study have been used in chemical analyses and extractions of non-polar substances such as the ones found in the oil sludges. Three of the selected co-solvents were aliphatic (*n*-pentane, *n*-hexane, and one branched aliphatic compound, isooctane) and two cyclic hydrocarbons (cyclohexane and toluene). Pentane and hexane have red flags in the Environmental, Health and Safety (EHS) legislation (Henderson et al. 2011). The physicochemical properties of the co-solvents used in this study and their toxicity status are shown in Table S1. The Hansen solubility parameter (HSP) (Hansen 2007) is a commonly used solubility parameter to predict the dissolution of a specific material into another one (Andecochea Saiz et al. 2018). These parameters can be used to explain the behaviour of the solvents in the oil recovery process (Zhao et al. 2017). The HSP values for the co-solvents used in this study are shown in Table S1.

Most of the studies about the treatment of oil sludges have been focused on crude oil tank bottom sludge (Hu et al. 2013; Mansur et al. 2016). However, oil sludges can also be found in other sources such as oil-water separators, desalinators, industrial wastewater, and from residuals after washing pipes in the petroleum industry facilities (da Silva et al. 2012; Hu et al. 2013; Egazar’yants et al. 2015). Therefore, there is a need to test the washing in oil sludges from different sources, so four different types of sludges were chosen in this study. The selected samples were an oil drilling, oil refinery, and two waste engine oil sludges generated in a tank by gravitational settling and centrifugation.

This study included four synthetic surfactants (Triton X-100, sodium dodecyl (SDS), Tween 80, and Triton X-114) and one biosurfactant (rhamnolipid). These surfactants have been used before for oil recovery purposes. Since the adsorption of the surfactant onto the sludge particles is not convenient for oil recovery (Wesson and Harwell 2000) and the oil sludge tends to be negatively charged, cationic surfactants were not considered in this study. Also, the surfactant adsorption is not beneficial for oil recovery purposes because it can reduce the surfactant concentration affecting the reduction of interfacial tension in the oil recovery (Barati et al. 2016). Moreover, anionic surfactants are more used in soil washing studies than cationic surfactants. Also, the latter are commonly less benign to the environment than other surfactants (Mao et al. 2015).

The aims of this study were to test the effects of different co-solvents with various degrees of toxicity (toluene, cyclohexane, hexane, pentane and isooctane) in the ORR, and to evaluate the effect of three important OSW factors (i.e. surfactant type and concentration, and surfactant to oil sludge (S/OS) ratio) in the ORR from four types of oil sludges.

## Materials and methods

### Oil sludges

An oil drilling sludge (ODS), two waste engine oil sludges obtained from two metal removal processes, gravitational settling (STS) and centrifugation (RS), and an oil refinery sludge (NSC) were analysed. An oil-water separator sludge (WSS) was used in the co-solvent selection for the oil sludge washing of the abovementioned sludges. This sludge was used in a previous study (Ramirez and Collins 2018). The oil sludges were sampled in the United Kingdom and had semi-solid states at room temperature. Table S2 shows the physicochemical characteristics of all sludges which were assessed in a previous study (Ramirez et al. 2019).

### Oil sludge washing (OSW)

The oil sludge, surfactant and the co-solvent were added to a 40-ml vial. Rhamnolipid and SDS were obtained from AGAE Technologies (Corvallis, Oregon, USA) and BDH Laboratory supplies, respectively. Tween 80, Triton X-114, and Triton X-100 were supplied by Sigma-Aldrich. The surfactants were kept in stock ultrapure water (18.2 MΩ·cm) solutions as follows: 10% (v/v) of Tween 80, Triton X-100 and Triton X-114, and 10% (w/v) of SDS and rhamnolipid. Table S3 and Table S4 show the CMC values and micelle sizes of these surfactants, respectively. These data were obtained in a previous study (Ramirez and Collins 2018). Due to the wide inter-surfactant variation of CMC, the absolute surfactant concentrations were expressed in terms of the critical micelle concentrations (xCMC) as suggested by Deshpande et al. (1999). An orbital shaker was used to agitate the vials for 1 h at 250 rpm. The vials were left for 12 h for gravitational separation purposes. A top layer of oil and co-solvent, a middle layer of water and surfactant, and the bottom layer of sediments were then observed. The co-solvent was evaporated with a gentle nitrogen stream, and the recovered oil was weighed. The oil recovery rate (ORR, %) was calculated with the masses of the recovered oil over the oil sludge (Zubaidy and Abouelnasr 2010; Hu et al. 2015).

### Screening of co-solvents in the oil sludge washing

Two synthetic surfactants, Triton X-100 and Triton X-114 (Sigma-Aldrich, UK), and a biosurfactant, rhamnolipid (AGAE Technologies, Corvallis, Oregon, USA)], were chosen for the co-solvent selection. Each surfactant was added at a 1:1 S/OS ratio and 2CMC because these surfactants had the maximum ORR values at this ratio and concentration in a previous OSW study with an oil-water separator sludge, WSS (Ramirez and Collins 2018).

A full-factorial experimental design was used. Three factors were included: Surfactant type (Triton X-100, Triton X-114, rhamnolipid), co-solvent (*n*-pentane, *n*-hexane, toluene, cyclohexane, and isooctane; high-purity, HPLC grade, Fisher Scientific) and co-solvent to oil sludge ratio, C/OS, (1:1, 2:1). The response variable was ORR (%). A total of 30 experimental runs in triplicate were analysed. A three-way ANOVA was used with the effect of the three factors. Paired t-tests (α=0.05) were performed for comparison of the means between co-solvents. Minitab 17.3.1 (Minitab Inc.) was used for the statistical analyses.

### Effect of the oil sludge washing (OSW) parameters in the oil recovery rate (ORR)

Two-stage experiments were completed, the S/OS ratio effect and the surfactant concentration effect. For the first stage, two ratios (1:1 and 5:1) were considered to test the S/OS ratio effect. The surfactant concentrations were selected from a previous study (Ramirez and Collins 2018). These concentrations were 1CMC for Triton X-100, 4CMC for Tween 80, 2CMC for rhamnolipid, 2CMC for Triton X-114, and 0.5CMC for sodium dodecyl sulphate (SDS); these concentrations gave the highest ORR values in each case (Ramirez and Collins 2018). The co-solvent to oil sludge (C/OS) ratio was 1:1. The data were analysed with a three-way analysis of variance with effects for the S/OS ratio, the sludge and surfactant types. A *post-hoc* Tukey’s test was performed to elucidate differences among the treatments.

In the second stage, the factors of the surfactant concentration effect were the oil sludge type (ODS, STS, RS, and NSC), surfactant type (Triton X-100, Tween 80, rhamnolipid, Triton X-114, and SDS) and surfactant concentration (0.5 CMC, 1CMC, 2CMC, 5CMC). A D-optimal experimental design was done to analyse these multi-level factors by a computer algorithm and a model (JMP®, Version 12.1, SAS Institute Inc., Cary, NC, 1989-2007). The input data for this model was taken from a preliminary study (Ramirez and Collins 2018). This experimental design uses an optimality criterion which decreases the generalized variance of the factor estimates in the pre-specified model (NIST 2013). Consequently, the predicted response has less uncertainty (de Aguiar et al. 1995). Also, the optimality criterion considers precise estimates of the coefficients in the pre-specified model (JMP 2013). Finally, the software detects the most suitable design which has the highest D-efficiency (%), and this value is obtained from the generalized variance (NIST 2013).

A three-way analysis of variance and a *post-hoc* Tukey’s test (α = 0.05) were done to test the effect of the surfactant concentration, and sludge and surfactant types in the ORR data. Furthermore, a control with no surfactant solution (i.e. ultrapure water only, 18.2 MΩ·cm) was done to compare with the surfactant solution data using a paired t-test (α = 0.05). The statistical analyses were executed with Minitab 17.3.1 (Minitab Inc.).

### Extractable petroleum hydrocarbons (EPH) extraction, clean-up, and separation of aliphatic and aromatic hydrocarbons of the recovered oil

The recovered oil (1 g) from the surfactant concentration effect experiment was added into a 22 ml glass vial with 10 ml of acetone:hexane (1:1, v/v) solution. The blank was 1 g of ultrapure water (18.2 MΩ·cm) and sand (50-70 mesh particle size). The vial was sonicated for 15 min at a frequency of 38 kHz to separate the sediment particles and release the EPH compounds. The sample was then shaken with a Stuart roller mixer SRT9D (Bibby Scientific Ltd.) for 60 min at 60 rpm. Deionized water (4 ml) was added to the vial, and it was frozen at -25°C to isolate the hexane. The hexane was then evaporated to 1 ml with nitrogen at 40°C. The samples were finally diluted (1:10) in hexane before the chromatographic analysis.

Gas chromatography grade silica gel (60 Å; 63 – 200 μm), anhydrous sodium sulphate (Fisher Scientific), and sand (50-70 mesh particle size) (Sigma-Aldrich) were activated and used as sorbents for the solid phase extraction (SPE) clean-up and separation of aliphatic and aromatic compounds. Silica gel (1 g), 0.5 g of anhydrous sodium sulphate and 1 g of sand were added consecutively to a 6 ml-polypropylene SPE cartridge with a 20 μm-polyethylene frit (Supelco), which was attached to a Visiprep™ vacuum manifold (Supelco) at a pressure of 254 mmHg. The cartridge was conditioned with hexane, and the sample (0.5 ml) was then added. The aliphatic and aromatic fractions were eluted successively with 3.5 ml of hexane and 9 ml of 3% of isopropanol in a hexane solution. The eluents were then evaporated to 1 ml with a nitrogen stream at 40°C.

Samples were analysed with an Agilent 6890 gas chromatograph-flame ionization detector (GC-FID). An SPB-5 GC capillary non-polar column (Sigma-Aldrich) was used. Sample (1 μl) was injected in a splitless mode. The air and hydrogen flows were 400 ml·min^-1^ and 30 ml·min^-1^, respectively. The make-up gas was nitrogen (25 ml·min^-1^) and the carrier gas was helium (3 ml·min^-1^). The temperatures of the detector and the inlet were set at 320°C and 285°C, respectively. First, the oven temperature was set at 60°C for 1 min, then ramped to 290°C at 8°C·min^-1^, and finally held for 6.75 min. The running time was 36.5 min (MADEP 2004). The calibration standards were EPH aliphatic hydrocarbons and polynuclear aromatic hydrocarbons mixes (Sigma-Aldrich). The OpenLab CDS Chemstation Edition software (v. C.01.07, Agilent Technologies) was used to analyse the chromatograms. The C_10_-C_18_ and C_19_-C_36_ aliphatic, and C_11_-C_22_ aromatic hydrocarbons fractions were then calculated, and a total EPH concentration was finally obtained. A two-way analysis of variance was done to test the effects of the sludge type and fractions of hydrocarbons in the total EPH concentration using Minitab 17.3.1 (Minitab Inc.).

## Results and discussion

### Selection of the co-solvent for the oil sludge washing

**Fig. 1:**
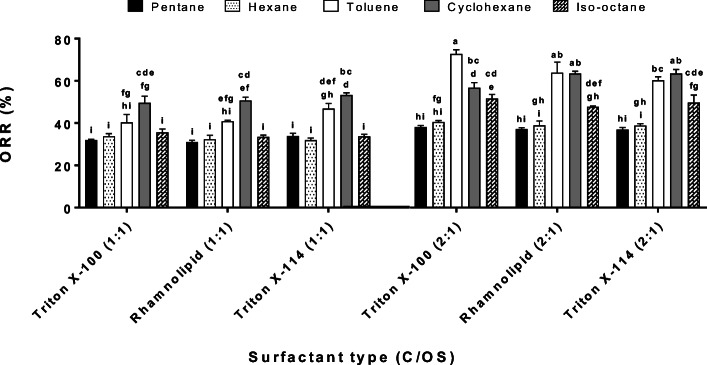
Oil recovery rates (ORR, %) with Triton X-100, rhamnolipid, Triton X-114 (2CMC and 1:1 S/OS) and co-solvents (1:1 and 2:1 C/OS ratios) from the washing of an oil-water separator sludge (WSS). Values with identical letters are not significantly different (Tukey's test, *p* > 0.05). The bars represent the standard error of the mean (SEM) (*n* = 3)

Figure 1 shows the ORR with the surfactants (2CMC, 1:1 S/OS) and co-solvents at 1:1 and 2:1 C/OS ratios.

The co-solvent type and C/OS ratio factors were highly significant (*p* < 0.01), whereas the surfactant type did not have a significant effect on the ORR (*p* = 0.396). The ORR values were higher at 2:1 than 1:1 C/OS ratio, and the ORR in pentane, hexane and isooctane did not change significantly between C/OS ratios (Fig. 1). The highest ORR values were found when toluene was used as co-solvent (2:1 C:OS) with Triton X-100 (73% ± 4) and rhamnolipid (64% ± 9). Also, cyclohexane had high ORR values at 2:1 C/OS ratio with Triton X-114 (63% ± 3) and rhamnolipid (63% ± 2). These values are higher compared to other studies (Biceroglu 1994; Avila-Chavez et al. 2007; Zubaidy and Abouelnasr 2010; Hu et al. 2015; Hu et al. 2017; Nezhdbahadori et al. 2018). These authors reported ORR values lower than 60%. An exception is El Naggar et al. (2010) that reported an oil recovery rate of 76% using toluene. Hu et al. (2016) reported that cyclohexane had a higher oil recovery (63.7%) compared to ethyl acetate (35.2%) and methyl ethyl ketone (34.8%) in a mechanical shaking extraction of oil sludge for 60 min at 250 rpm. Since only co-solvents were used in the abovementioned studies, these results could elucidate the important role of surfactants in the enhancement of the oil recovery.

Low ORR values were found at 1:1 C/OS ratio because the volume of co-solvent was not probably enough to extract the oil (i.e. saturation of the co-solvent by the oil) that was recovered by the action of the surfactant. Also, Kamal and Khan (2009) showed that there was a saturation of the co-solvent by the crude oil at low C/OS ratios, and this event gave lower oil recovery values compared to high C/OS ratios. In contrast, oil solubility in the co-solvent can be improved at higher C/OS ratios, so the ORR is high (Zubaidy and Abouelnasr 2010; Al-Zahrani and Putra 2013; Hu et al. 2015; Hu et al. 2017). Therefore, higher C/OS ratios than 2:1 should be explored in future studies to confirm if the ORR improves and it is cost-effective.

The ORR with cyclohexane was not significantly different from toluene in all C/OS ratios and surfactant type combinations (*p* = 0.62), except for rhamnolipid at 1:1 and Triton X-100 at 2:1 C/OS ratios (*p* = 0.026 and *p* = 0.037). Toluene and cyclohexane had the highest ORR values in this study as shown before. However, toluene is less benign to the environment and more harmful to human health than cyclohexane. Therefore, cyclohexane can be an alternate co-solvent to toluene in the OSW process. Young (2007a) mentioned that cyclohexane has moderate toxicity (2 of 4), and the 11th Annual Report on Carcinogens of the National Toxicology Program (NTP 2005) and Guerra et al. (2017) reported that cyclohexane is not considered to be carcinogenic (Table S1). Hu et al. (2015) indicated that cyclohexane can be an appropriate solvent for oil recovery (41% ORR during 30 min of extraction at 4:1 C/OS) compared to dichloromethane, methyl ethyl ketone, and ethyl acetate (30% ORR for these co-solvents).

Table S1 shows the physicochemical properties of the co-solvents which can elucidate the reasons for the different ORR values, and one of these properties is the molecular weight. In fact, Rincón et al. (2005) informed that the solvent molecular weight and oil recovery yields have positive proportional linearity due to a reduction in the solubility difference between the solvent and solute. Toluene has a higher molecular weight (92.14 g·mol^-1^) and higher ORR values than hexane (86.18 g·mol^-1^) and pentane (72.15 g·mol^-1^) which had low ORR values. Moreover, isooctane had the highest molecular weight (114.23 g·mol^-1^), and it had a higher ORR value at 2:1 C/OS ratio compared to hexane and pentane. However, the ORR values of isooctane were generally lower than cyclohexane and toluene. This finding suggested that there are probably other physicochemical properties of the solvents that could influence the oil recovery such as the Hansen solubility parameter (isooctane had the lowest HSP value, 14.3 MPa^½^).

The Hansen Solubility Parameter (HSP) could explain the differences among the ORR values of the co-solvents used in this study. In fact, cyclohexane and toluene had the highest reported HSP values (Table S1) and the highest ORR values (Fig. 1). Conversely, pentane, hexane, and isooctane had the lowest oil recoveries (<40%) and low HSP values. The HSP value has been successfully used to predict the solubility and can explain the behaviour in the oil recovery after a solvent extraction process from different matrices (Khor et al. 2017; Zhao et al. 2017; Casalini et al. 2018). For the case of surfactants, these HSP calculations can be more complicated due to the interaction of the surfactant amphiphilic structure with the oil sludge. Nevertheless, there are recently some reports that predicted these solubility parameters by Molecular Dynamics (MD) simulations (Faasen et al. 2020). Indeed, future studies can also focus on the calculations of HSP values for surfactant adsorption onto semi-solid matrices (e.g. oil sludge) both experimentally and theoretically by MD. Then, these findings can contribute to the selection of surfactants for oil recovery purposes.

Even though high C/OS ratios tend to favour high ORR values, the 1:1 C/OS ratio was selected due to logistic reasons. The reason was that the WSS sample was completely used in previous studies (Ramirez and Collins 2018; Ramirez et al. 2019), and the OSW parameters (S/OS ratio, surfactant concentration and type) were analysed with a fixed 1:1 C/OS ratio. Therefore, it was decided to use the 1:1 C/OS with the other sludges in this study, so the results could be compared with the WSS sample. The 2:1 C/OS data was shown in this study to indicate that higher C/OS ratios (e.g. 2:1, 3:1, 5:1 C/OS) should be considered in future studies to check if this improvement in the oil recovery can be cost-effective. Nevertheless, the main objective of this experiment was to test alternative co-solvents to toluene that are more benign for the environment. Therefore, cyclohexane was the co-solvent chosen for the oil sludge washing in the S/OS ratio and surfactant concentration effects experiments.

### Effect of the S/OS ratio in the oil recovery from different types of oil sludges

**Fig. 2:**
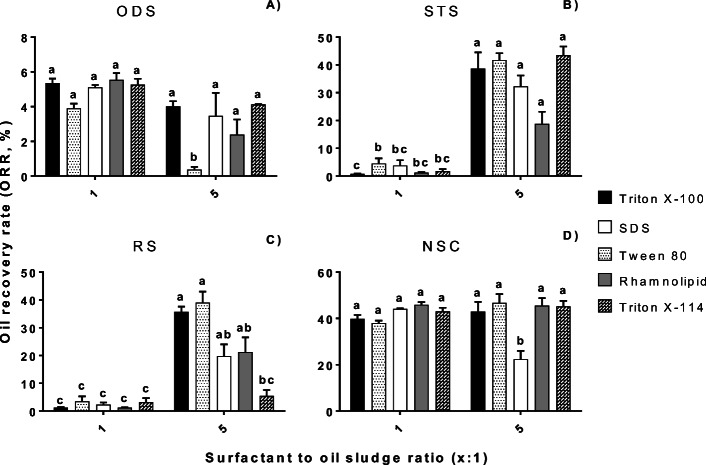
Oil recovery rates (ORR, %) from four types of oil sludges, **a** ODS, **b** STS, **c** RS, and **d** NSC at 1:1 and 5:1 S/OS ratios. Cyclohexane was used as a co-solvent (1:1 C/OS ratio). A Tukey's test compared the S/OS ratios with surfactants per oil sludge. Values with the same letters are not significantly different (*p* > 0.05). The bars indicate the standard error of the mean, SEM (*n* = 3)

The effects of S/OS ratio and surfactant type on the oil recovery varied in the different types of oil sludge (Fig. 2).

The three-way ANOVA showed a highly significant effect of the S/OS ratio, and surfactant and oil sludges types on the ORR (*p* < 0.01). Consequently, there was a highly significant effect for both S/OS ratio and oil sludge type factors in the oil recovery (*p* < 0.01) whereas the surfactant type was the only non-significant factor (*p* = 0.651). A *post-hoc* Tukey’s test (α = 0.05) indicated that the oil sludges were significantly different among them, except for RS and STS. It was expected this similarity in the ORR values for these two sludges because they originated from the same source (i.e. waste engine oil). The only difference was the metal removal treatment done for both sludges as mentioned before in “Oil sludges” section. Further detailed analyses of the oil sludges such as particle size distribution and SEM micrographs during the oil recovery process can be performed to elucidate the reasons of these substantially low ORR at this low S/OS ratio. For instance, a migration behaviour study of oil and solids in oil sludge during the oil recovery process can be performed as Wang et al. (2017) did in their oil sludge centrifugation study.

Overall, Tween 80 and Triton X-100 had the highest ORR values in all sludges. ODS (Fig. 2a) had highly significant ORR at 1:1 S/OS ratios (*p* < 0.01). A Tukey’s test (α = 0.05) showed that the ORR value from ODS using Tween 80 (5:1 S/OS ratio) was significantly lower (0.37% ± 0.28) than the ORR values of the other surfactants (2% – 5%) (Fig. 2a). Also, a previous study found high ORR values at low S/OS ratios in an OSW process of an oil-water separator sludge (Ramirez and Collins 2018). Recently, Ren et al. (2020) reported the lowest residual oil rate at a low S/OS ratio (2.5:1) in a washing process with a biosurfactant of highly-viscous oil sludge. However, it is commonly reported a high ORR at high S/OS ratios (Peng et al. 2011; Wu et al. 2012). This decrease in the oil recovery at high rates could be due to the washing time that was not enough to reach the thermodynamic equilibrium to recover all the oil from this type of oil sludge at S/OS high ratios. Therefore, this oil sludge only needed a low S/OS ratio to reach the equilibrium and recover the maximum volume of oil with less surfactant solution as reported in other studies (Zubaidy and Abouelnasr 2010; Ramirez and Collins 2018). Moreover, ODS had the lowest oil content and the highest solid content, 1 and 86 %, respectively (Table S2). Since the ORR values for ODS tended to be higher at 1:1 than 5:1 S/OS ratio, all the oil could be recovered at this low S/OS ratio. STS (Fig. 2b) and RS (Fig. 2c) had highly significant ORR values at 5:1 than 1:1 S/OS (*p* < 0.01). The ORR values from NSC were not significantly different between both S/OS ratios (*p* = 0.095) (Fig. 2d), except for SDS (5:1 S/OS). Particularly, this value was significantly lower (22% ± 6) than the other surfactants (38% – 47%).

The S/OS ratios with the highest ORR values per oil sludge (Fig. 2) were then used for the second experimental stage to assess the surfactant concentration effect in the oil sludges.

### Effect of the surfactant concentration in the oil recovery from different types of oil sludges

**Fig. 3:**
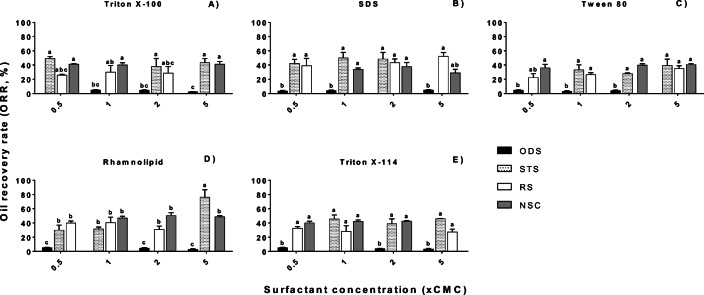
Oil recovery rate (ORR %) from all oil sludges at different surfactant concentrations (0.5, 1, 2, and 5 CMC). The S/OS ratios were selected from Fig. 2. The co-solvent was cyclohexane (1:1 C/OS ratio). A Tukey's test compared the surfactant concentrations with oil sludges per surfactant. Values with the same letters are not significantly different (*p* > 0.05). The bars indicate the standard error of the mean, SEM (*n* = 3)

For this second stage, the experimental design model with the highest D-efficiency (89.91%) and the lowest average variance of the prediction (0.94) was chosen. The S/OS ratios for each sludge were selected according to the findings from the S/OS ratio effect experiment (See “Effect of the S/OS ratio in the oil recovery from different types of oil sludges” section) where it was established that the highest ORR were obtained at 1:1 for ODS and 5:1 for STS, RS, and NSC. Even though NSC had no significant differences in the oil recovery at both ratios, the 5:1 ratio was selected because most of the studies reported higher recoveries at S/OS higher ratios. The effect of surfactant concentrations in the ORR values from different types of oil sludges is shown in Fig. 3.

There were highly significant differences in the sludge and surfactant types (*p* < 0.01), but there was no effect of the surfactant concentration in the ORR values (*p* = 0.745). The *post-hoc* test (α = 0.05) showed differences among the surfactants in each oil sludge. The highest ORR values in each sludge were 76% (± 18) with rhamnolipid–5CMC in STS, 52% (± 9) with SDS–5CMC in RS, 51% (± 6) with rhamnolipid–2CMC in NSC, and 5% (± 0.87) and 5% (± 0.77) with rhamnolipid and Triton X-114 at 0.5CMC in ODS, respectively (Fig. 3). These results showed that the oil recovery could be favoured by an oil mobilization phenomenon in the case of ODS (surfactant concentration below CMC) whereas the oil solubilization into the surfactant micelles (above CMC) enhanced the oil recovery in the other sludges.

The zeta potential is also related to the surfactant concentration due to the formation of the electrical double layer at the oil-water interface. The zeta potential decreases at higher surfactant concentrations and then it tends to reach a plateau. This phenomenon can be due to the formation of micelles and full saturation of surfactant monomers (Kumar and Mandal 2018). Also, high surfactant concentrations contribute to the dominance of the electrical double layer which diffuses the surface charge away by the electric field of the layers (Gray-Weale and Beattie 2009). Consequently, the surfactant reduces both the interfacial tension (IFT) and zeta potential. Finally, when the surfactants cover all the oil droplets in the sludge at higher concentrations, there is no further effect by increasing the surfactant concentration (Deng et al. 2002). Indeed, these facts can also explain the higher ORR obtained at low surfactant concentrations in our study. In the case of STS and RS, it can be possible that the zeta potential decreased more until stabilization at 5CMC when the highest ORR occurred for both sludges. Therefore, it is recommended in future studies to measure this parameter as it could also be important for surfactant selection purposes.

**Fig. 4:**
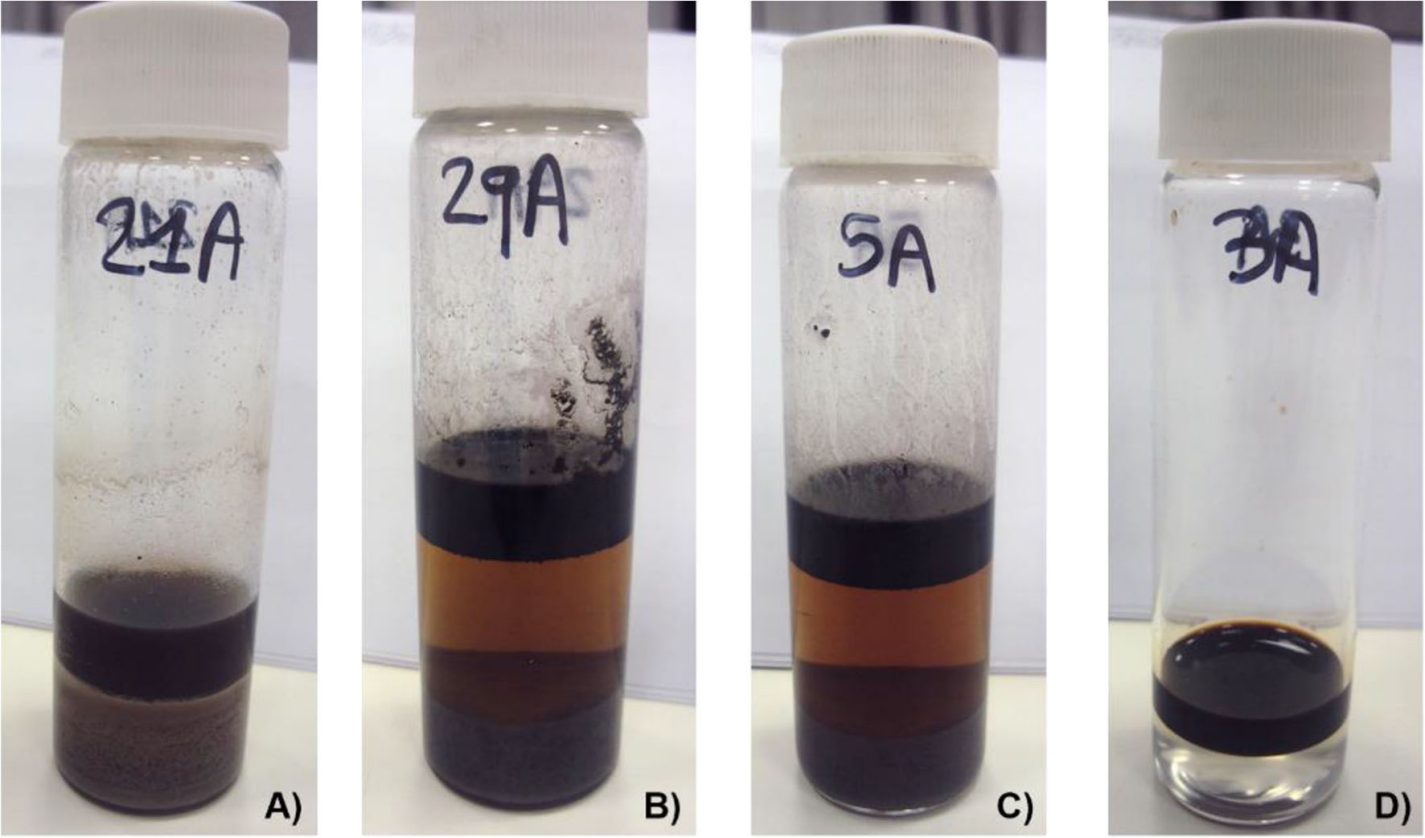
Vials with the final separation of the three layers (from top to bottom: oil and cyclohexane, water and surfactant, and sediment) obtained after the oil sludge washing (OSW). **a** ODS with Tween 80–0.5 CMC. **b** STS with Triton X-100–2 CMC. **c** RS with Triton X-114–0.5 CMC. **d** NSC with rhamnolipid–1 CMC. All S/OS ratios used were 5:1, except for (**a**) which was 1:1

Pictures of the three layers observed at the end of the OSW process of all sludges are shown in Fig. 4.

After the OSW, the ODS sample showed more sediment in the bottom layer compared to the other sludges (Fig. 4a) because this was an oil drilling sludge and its solid content was the highest with 86% (±0.11). On the contrary, the lowest amount of sediment material was observed in the NSC sludge (Fig. 4d) due to its low solid content (1% ± 0.07) as shown in Table S2. The separation of layers in RS and STS was difficult to achieve because it was found water and solid remnants in the top layer (Fig. 4b and c). This event could be due to a strong W/O emulsion present in these sludges, where the solids can be either absorbed in the interface and/or dispersed in the oil and water parts of the emulsion (Duan et al. 2019). Therefore, the top layer was left further overnight to ensure complete gravitational separation of the water and sediment traces. However, when NSC was washed with rhamnolipid, the top oil layer had no visual presence of sediments (Fig. 4d).

Also, Hu et al. (2015) reported the presence of water remnants in the top oily layer. They mentioned that although this presence of water could overestimate the ORR values (also measured by weight by them), this event only had a minimal influence on the overall ORR values due to the equal treatment in all samples (Hu et al. 2015). Therefore, their results were comparable. Certainly, this was not the exception in the present study because the samples were prepared following the same protocol, and the same proportion of oil sludge was fixed throughout the study (i.e. variable volumes of surfactant solution in one part of sludge). In addition, the water found in the top layer was negligible compared with the amount of recovered oil.

When RS and STS were washed with rhamnolipid, it was observed that the recovered oil was more viscous and had no visual indication of water possibly because the rhamnolipid broke the emulsion in these sludges. Long et al. (2013) reported the demulsification potential of rhamnolipids in the emulsion breaking of waste crude oil, so this process was able to remove about 90% of water. Sha et al. (2012) reported that the emulsion breaking feature of rhamnolipids could be linked with the high surface activity of this biosurfactant.

Also, when SDS (e.g. 2CMC) and rhamnolipid (0.5CMC and 2CMC) were used to wash the RS and ODS samples, respectively, the top oily layer was separated. Also, this was the case when rhamnolipid (Fig. 4d) and Triton X-114 were used to wash the NSC sludge. Consequently, an additional gravitational separation was not necessary. However, it was found some sediment traces in the recovered oil from the SDS–5CMC–ODS and SDS–NSC samples.

Table 1 showed the comparison of the ORR values between the OSW controls with no surfactant and the highest ORR values from the surfactant concentration effect experiment. In addition, the ORR values from an oil-water separator sludge (WSS), used in the co-solvent selection, were considered.

**Table 1:** Comparison of the oil recovery rate (ORR %) mean values between the control (only water) and surfactant-treated samples from the OSW process

Sample	ORR% (water = 0 CMC)	ORR% (with surfactant solution) ^a^	*p*-values ^b^ (H_1_: μ_d_ > 0)
ODS	6 (± 0.15)	Rhamnolipid (0.5CMC) = 5% (± 0.87)	0.847
STS	60 (± 8)	Rhamnolipid (5CMC) = 76% (± 18)	0.132
RS	49 (± 2)	SDS (5CMC) = 52% (± 9)	0.749
NSC	59 (± 7)	Rhamnolipid (2CMC) = 51% (± 6)	0.795
WSS ^c^	22 (± 1)	Triton X-114 (2CMC) = 53% (± 2)	<0.01

All ORR are mean values with the standard deviation (*n* = 3)

^a^ The ORR values were the highest in the surfactant concentration experiment (Fig. 3) and the S/OS ratios were 1:1 for ODS and 5:1 for STS, RS, and NSC (Fig. 2). Cyclohexane was used as a co-solvent (1:1 C/OS ratio)

^b^ The alternative hypothesis (H_1_) checked if the difference (μ_d_) between ORR mean values was higher than 0

^c^ The ORR data for WSS was taken from Ramirez and Collins (2018)

A paired t-test (α = 0.05) showed that the ORR values of the controls with no surfactant and cyclohexane as the co-solvent were not significantly different to the values when the surfactant was used (Table 1), except for WSS. In this sludge, the ORR value with surfactant was significantly higher than the control (*p* < 0.01). In general, it has been reported an improvement in the oil removal in multiple soil washing studies with the addition of surfactants (Deshpande et al. 1999; Urum et al. 2004; Urum et al. 2006; Peng et al. 2011; Wu et al. 2012). However, some studies reported similar oil removal rates from the soil in the treatments with and without surfactant (Bhandari et al. 2000; Urum and Pekdemir 2004; Hernández-Espriú et al. 2013).

A previous study characterized the surfactants used in this study, so the CMC (pendant drop method), micelle size (dynamic light scattering, DLS), and the surface activity (oil displacement test) were determined (Ramirez and Collins 2018). In general, it was found that rhamnolipid, Triton X-114, and Triton X-100 had lower CMC values and higher micelle sizes, surface activities, and surface tension reduction compared to Tween 80 and SDS (Table S3 and Table S4). These attributes of the former surfactants can also explain the higher ORR values found in this study compared to the latter. For instance, rhamnolipid was the surfactant with the highest ORR, 76% (Table 1). In fact, these features of low surface (air/water)/interfacial (oil-water) tensions are preferred in the petroleum industry for oil recovery-enhancement purposes (Austad and Milter 2000), and rhamnolipids are characterized for having these features (e.g. low CMC and high surface activity) and they are known to be more benign to the environment than synthetic surfactants (Liu et al. 2018b).

Micelle sizes are related to the micellar aggregation number established by the number of surfactant monomers per micelle. Therefore, bigger micelles can solubilize more oil inside their hydrophobic cores increasing their aggregation number (Rosen and Kunjappu 2012). Consequently, the oil recovery can be improved. Therefore, solubilization of oil hydrocarbons at concentrations higher and equal than CMC tend to be high for non-ionic surfactants (Li et al. 2016), so these surfactants had the highest ORR in this study.

The results obtained in this study and the high effect of the S/OS ratio and the surfactant type in the ORR values in all oil sludges suggested that it is necessary to perform a bench-scale study of an oil sludge sub-sample before treatment at a large scale. By doing this, it can be determined if the surfactants and a high S/OS ratio are necessary for the washing process.

### Extractable petroleum hydrocarbons (EPH) concentrations in the recovered oil

**Fig. 5:**
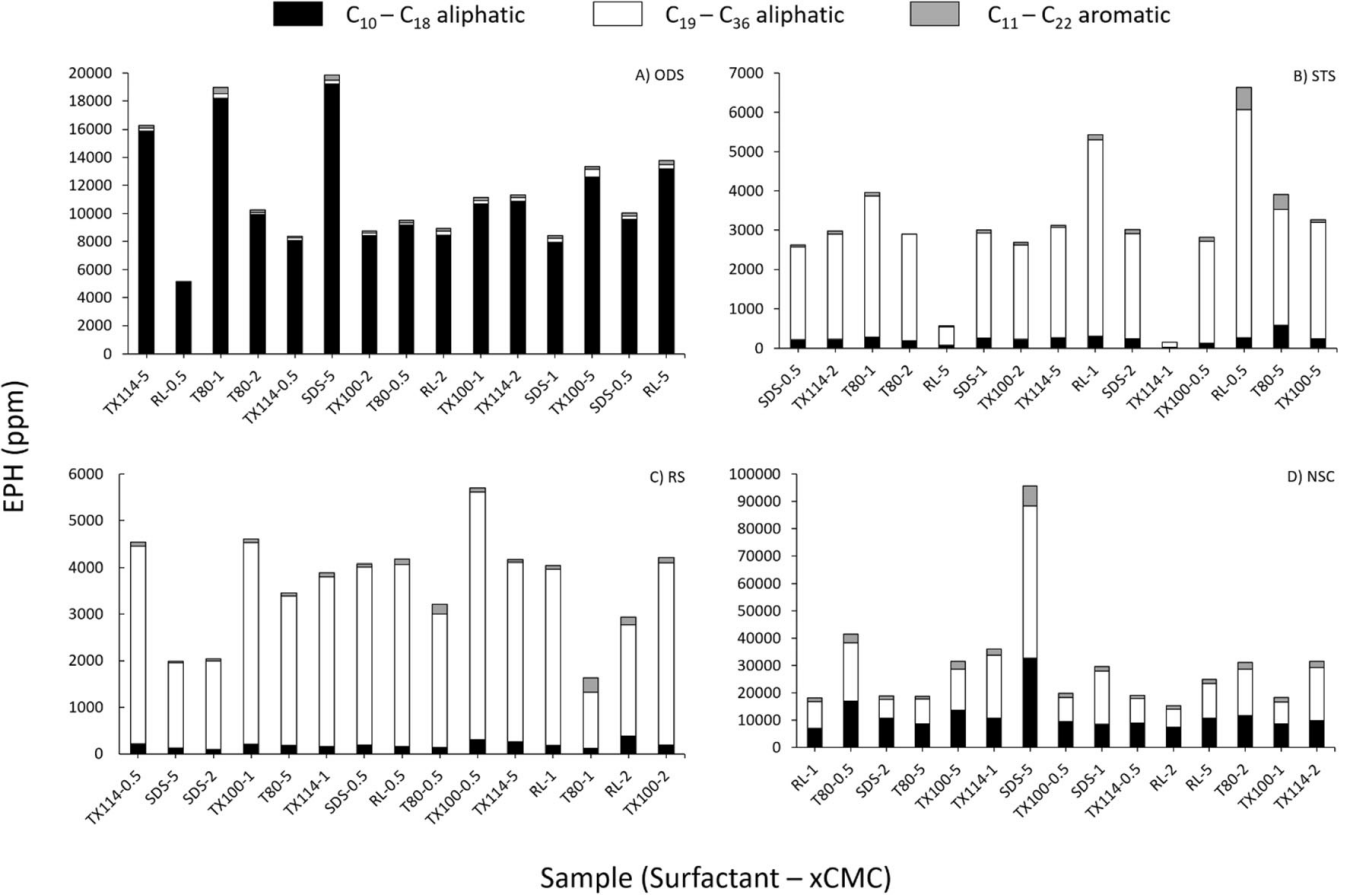
Aliphatic and aromatic extractable petroleum hydrocarbons (EPH) concentrations of the recovered oil from four oil sludges: **a** ODS, **b** STS, **c** RS, and **d** NSC. Five surfactants, Triton X-114 (TX114), rhamnolipid (RL), Tween 80 (T80), Triton X-100 (TX100), and sodium dodecyl sulphate (SDS) were used in the washing at different concentrations (0.5, 1, 2, 5 CMC)

The goal of this analysis was to detect the distribution of the aliphatic and aromatic oil hydrocarbon fractions concentrations in the recovered oil at varying concentrations. Figure 5 shows the EPH concentrations in the recovered oil from the four types of oil sludges.

A one-way ANOVA showed that the oil sludge type had a highly significant effect on the total EPH concentrations (*p* < 0.01), but there were no significant differences in the surfactant type (*p* = 0.946) and surfactant concentration (*p* = 0.808). The inter-surfactant and inter-sludge differences in the concentrations of aliphatic and aromatic oil hydrocarbons indicated that it is important to evaluate different surfactant formulations (Fig. 5) before choosing an optimal OSW process. In this study, the surfactant formulations that recovered the highest EPH concentrations in each sludge were the following: For ODS were Triton X-114 (5CMC), Tween 80 (1CMC), and SDS (5CMC); for STS was rhamnolipid (0.5 and 1CMC), for RS was Triton X-100 (0.5CMC), and for NSC was SDS (5CMC). STS and RS had a high concentration of C_19_ – C_36_ aliphatic hydrocarbons, whereas ODS had a high concentration of light aliphatic hydrocarbons, C_10_ – C_18_. Ren et al. (2020) have also found light oil hydrocarbons compounds in the recovered oil.

The importance of these findings was to determine the potential reuse of the recovered oil as a feedstock for fuel production. For example, a recovered oil with high concentrations in the range of C_16_ – C_34_ oil hydrocarbon fractions can be used in the production of heavy fuel oil (Wang et al. 2003). On the contrary, if the recovered oil has a high concentration of light hydrocarbon fractions (C_10_ – C_18_), it can be reused in the production of diesel (Giles 2010; Zhao et al. 2018). Also, these data are important for toxicity reasons. For example, by assessing the aromatic fraction, polycyclic aromatic hydrocarbons (PAHs) can be determined because these compounds are considered to be genotoxic to humans, specifically PAHs with high molecular weights (Robertson et al. 2007).

Villalanti et al. (2006) stated that gas chromatography is a rapid method to assess of the oil hydrocarbons fractions, and this information can aid in the selection of crude oils with reuse potential. In addition, Hu et al. (2015) indicated that the oil quality can be assessed with the EPH concentrations from the GC profiles. However, this remark has to be cautiously considered because Giles (2010) mentioned that GC data cannot measure directly the quality of the oil, and the sample has to be fractionated by distillation methods to confirm the quality. Therefore, the use in this study of the GC data is not considered to be a complete validation of the oil quality, but it was considered to establish the potential reuse of the oil in the fuel production. Furthermore, other tests such as the pour and flash point, the heat of combustion, API gravity, and sulphur content can evaluate directly the quality (Abouelnasr and Zubaidy 2008; Zubaidy and Abouelnasr 2010; Hu et al. 2015).

## Conclusions and further recommendations for the oil sludge washing

The main aim of the co-solvent effect experiment was to select a more benign-to-the-environment co-solvent than toluene. First, it was found higher ORR values at 2:1 C/OS ratio than 1:1. Particularly, the ORR data from this study (about 75%) were higher compared to other studies (<60%). Since these studies used solvent extraction with no surfactants, the important role of surfactants in the oil recovery was evidenced in the present study. Moreover, this study showed the differential performance of the co-solvents in the OSW, and the differences in the ORR values can be explained by the HSP values of the co-solvents. For instance, cyclohexane and toluene had about a two-fold increase in the ORR from 1:1 to 2:1 C/OS ratio, whereas, in the case of pentane, hexane and isooctane, there were no significant changes in the ORR values between the C/OS ratios. In addition, cyclohexane had no significant differences than toluene in the ORR values. Since cyclohexane is less hazardous to the environment than toluene, it was chosen for the S/OS ratio and surfactant concentration effects studies. Although higher C/OS ratios (e.g. 2:1 C/OS) tend to favour the oil recovery, it was chosen the 1:1 C/OS ratio due to logistic purposes. Nevertheless, these data showed that higher C/OS ratios (e.g. 2:1, 3:1, 5:1 C/OS) can be considered in future studies to enhance the oil recovery if the application of higher ratios is cost-effective for the process.

This study also analysed the oil recovery in an OSW process of four types of oil sludges. The key results from this study are that the S/OS ratio had a highly significant effect on the oil recovery, and the surfactant concentration had no effect on the oil recovery. The surfactants with the highest ORR values were rhamnolipid, Triton X-100, and Triton X-114 (i.e. 40 – 70%). The S/OS ratio was dependent on the oil sludge. The results of the ODS sample showed that a high S/OS ratio does not guarantee a high recovery due to high surfactant volume, instead, a maximum oil volume can be recovered at low S/OS ratios in this type of oil sludge. Also, there was no significant difference in the ORR between the washing with and without surfactant solution in all the four oil sludges analysed in this study. Only the WSS sample, an oil-water separator sludge analysed in a previous study, had a highly significant ORR compared to the control.

Some general recommendations to perform the OSW in oil sludge samples can be suggested based on the main findings on the OSW experiments from this study. Initially, a bench-scale test can be performed to evaluate the ORR with and without surfactant solution at a low and high S/OS ratios (e.g. 1:1 and 5:1 S/OS). Rhamnolipid, Triton X-100 and Triton X-114 could be used as these were the surfactants with the highest oil recovery rates in this study. This first assay can help quickly to decide if a surfactant is necessary, and if a low S/OS ratio is enough to have a maximum oil recovery. If it is not required a surfactant or high S/OS ratio, costs can be saved. If a surfactant is required for the OSW, the additional value is the selective extraction of the oil hydrocarbon fractions. Therefore, the quality of the recovered oil can be improved, and it can be reused as fuel.

## Electronic supplementary material

ESM 1 (DOCX 36 kb)

## Abbreviations

C/OS: Co-solvent to oil sludge ratio
CMC: Critical micelle concentration
EPH: Extractable petroleum hydrocarbons
HSP: Hansen Solubility Parameter
NSC: Oil refinery sludge
ODS: Oil drilling sludge
ORR: Oil recovery rate
OSW: Oil sludge washing
RS: Waste engine oil sludge from centrifugation
SDS: Sodium dodecyl sulphate
S/OS: Surfactant to oil sludge ratio
SPE: Solid-phase extraction
STS: Waste engine oil sludge from gravitational settling
WSS: Oil-water separator sludge.
