# Supplementary material for: Oil sludge washing with surfactants and co-solvents: oil recovery from different types of oil sludges
Source: Environ Sci Pollut Res Int. 2020 Sep 25;28(5):5867–79. doi: 10.1007/s11356-020-10591-9 (PMC7838146; doi:10.1007/s11356-020-10591-9)
Supplement: Supplementary file 1 — (DOCX 36 kb) [file 11356_2020_10591_MOESM1_ESM.docx]

# Oil sludge washing with surfactants and co-solvents: Oil recovery from different types of oil sludges

Diego Ramirez ^a,*^, Liz J. Shaw ^a^, Chris D. Collins ^a^

^a^ Department of Geography and Environmental Science, University of Reading, Reading, RG6 6DW, UK

* Corresponding author.

*E-mail addresses:* [diego.ramirez.guerrero@gmail.com](mailto:diego.ramirez.guerrero@gmail.com) (D. Ramirez), [e.j.shaw@reading.ac.uk](mailto:e.j.shaw@reading.ac.uk) (L. J. Shaw), [c.d.collins@reading.ac.uk](mailto:c.d.collins@reading.ac.uk) (C.D. Collins).

Table S 1. Description of the co-solvents used in the study.

| **Co-solvent** | **Formula** | **MW ^1^** | **Water solubility** | **log K_ow_ ^2^** | **Melting**  **point** | **HSP (δ) ^3^** | **Toxic properties and environmental impact ^4^** | | | | |
| --- | --- | --- | --- | --- | --- | --- | --- | --- | --- | --- | --- |
|  |  | ***g·mol^-1^*** | ***mg·l^-1^*** |  | ***°C*** | ***MPa^½^*** | **Waste** | **Environment** | **Humanhealth** | **Flammability** | **Reactivity** |
| ***n*-pentane** | C_5_H_12_ | 72.15 | 40 | 3.39 | -130 | 14.5 | 5 | 6 | 8 | 2 | 10 |
| ***n*-hexane** | C_6_H_14_ | 86.17 | 9.5 | 4.11 | -96 | 14.9 | 5 | 3 | 4 | 2 | 10 |
| **Toluene** | C_7_H_8_ | 92.14 | 520 | 2.7 | -95 | 18.2 | 6 | 3 | 4 | 4 | 10 |
| **Cyclo-hexane** | C_6_H_12_ | 84.16 | Immiscible | 3.44 | 6.47 | 16.8 | 5 | 5 | 7 | 2 | 10 |
| **Iso-octane** | C_8_H_18_ | 114.23 | Immiscible | 5.18 | -107 | 14.3 | 6 | 4 | 8 | 3 | 10 |

All physicochemical data were retrieved from the *ChemSpider database* <http://www.chemspider.com/> (Royal Society of Chemistry, 2016), except for the Hildebrand solubility parameters (HSP) of all solvents Hansen (2007).

**^1^** Molecular weight (MW).

**^2^** Octanol-water partition coefficient (K_ow_).

**^3^** Hansen solubility parameter (HSP): Hansen (2007).

**^4^** Solvent toxicity and environmental issues were established in the *GlaxoSmithKline (GSK) Solvent Selection Guide* on 2009 (Royal Society of Chemistry, 2010). Impact score from 1 to 3 (red; high impact) to 8-10 (green; low impact) (Henderson *et al.*, 2011). *Waste*: Recycling, incineration, volatile organic compounds (VOC), and biotreatment issues. *Environment*: Fate and effects. *Health*: exposure potential; acute and chronic effects on human health. *Flammability*: Storage and handling. *Reactivity*: Factors affecting the stability of the solvent (Henderson *et al.*, 2011).

Table S 2. Physicochemical properties of the oil sludges.

| Sludge |  | | Wet and dry contents | | | Extractable Petroleum Hydrocarbons (EPH) | | | |
| --- | --- | --- | --- | --- | --- | --- | --- | --- | --- |
|  | **Oil**  **(%)** | **Water**  **(%)** | **Wet content** | **Solid (as a part of the dry content)** | **Organic material (as a part of the dry content)** | **C_10_-C_18_ aliphatic (%)** | **C_19_-C_36_ aliphatic (%)** | **C_11_-C_22_ aromatic (%)** | **Total EPH**  **concentrations (ppm)** |
| ODS | 1 (±0.26) | 99 (±0.26) | 13 (±0.02) | 86 (±0.11) | 1 (±0.10) | 98 | 0.53 | 1.47 | 6,000 (± 145) |
| STS | 50 (±14) | 50 (±14) | 41 (±0.16) | 35 (±0.16) | 24 (±0.09) | 13 | 83 | 4 | 1,550 (± 506) |
| RS | 39 (±1) | 61 (±1) | 35 (±2) | 38 (±0.17) | 27 (±2) | 10 | 85 | 5 | 949 (± 392) |
| NSC | 88 (± 11) | 12 (± 11) | 60 (±2) | 1 (±0.07) | 39 (±2) | 69 | 30 | 1 | 68,000 (± 6,070) |

The oil sludges were analysed in a previous study. See Ramirez *et al.* (2019) for details.

Each result is the mean (*n* = 3) with the standard deviation is in parentheses.

Oil and water contents were determined by high-field nuclear magnetic resonance (NMR), EPH concentrations were determined by GC-FID, and wet and dry contents by the oven-drying method.

Table S 3. Critical Micelle Concentration (CMC) values of the surfactants measured by the pendant drop method ^1^.

|  |  | CMC (mM) | |
| --- | --- | --- | --- |
| Surfactant | **Surface tension ^2^ (mN·m^-1^)** | **Observed** | **Reported ^3^** |
| Rhamnolipid | 29 | 0.048 | 0.0092-0.42 |
| Tween 80 | 44 | 1.12 | 0.011 |
| Triton X-114 | 27 | 0.36 | 0.2-0.35 |
| Triton X-100 | 31 | 0.28 | 0.17-0.3 |
| SDS | 38 | 8.37 | 8.0 |

**^1^** These data were determined in a previous study (Ramirez and Collins, 2018).

**^2^** Surface tension of the water used in all surfactant solutions (74 mN·m^-1^).

**^3^** CMC values reported in other studies at 20-25°C: Tween 80 (Hillgren, Evertsson and Aldén, 2002), Triton X-100 and Triton X-114 (Arnold and Linke, 2007), rhamnolipid (Abalos *et al.*, 2001; Torres *et al.*, 2011), and SDS (Tadros, 2005).

Table S 4. Micelle sizes of the surfactants measured by dynamic light scattering (DLS) ^1^.

| Surfactant | Concentration (mM) | Micelle size (nm) ^2^ |
| --- | --- | --- |
| Rhamnolipid | 0.09 | 13.5 (±0.61) |
| Tween 80 | 3 | 12.1 (±0.17) |
| Triton X-114 | 1 | 14.58 (±0.006) |
| Triton X-100 | 1 | 7.62 (±0.05) |
| SDS | 14 | 3.61 (±0.06) |

**^1^** These data were determined in a previous study (Ramirez and Collins, 2018).

**^2^** The micelle size corresponded to the hydrodynamic diameter.

**REFERENCES**

Abalos, A., Pinazo, A., Infante, M. R., Casals, M., García, F. and Manresa, A. (2001) 'Physicochemical and Antimicrobial Properties of New Rhamnolipids Produced by Pseudomonas aeruginosa AT10 from Soybean Oil Refinery Wastes', *Langmuir,* 17(5), pp. 1367-1371.

Arnold, T. and Linke, D. (2007) 'Phase separation in the isolation and purification of membrane proteins', *BioTechniques,* 43(4), pp. 427-440.

Hansen, C. M. (2007) *Hansen Solubility Parameters: A User's Handbook.* Second Edition edn. Boca Raton, FL, USA: CRC press.

Henderson, R. K., Jimenez-Gonzalez, C., Constable, D. J. C., Alston, S. R., Inglis, G. G. A., Fisher, G., Sherwood, J., Binks, S. P. and Curzons, A. D. (2011) 'Expanding GSK's solvent selection guide - embedding sustainability into solvent selection starting at medicinal chemistry', *Green Chemistry,* 13(4), pp. 854-862.

Hillgren, A., Evertsson, H. and Aldén, M. (2002) 'Interaction Between Lactate Dehydrogenase and Tween 80 in Aqueous Solution', *Pharmaceutical Research,* 19(4), pp. 504-510.

Ramirez, D. and Collins, C. D. (2018) 'Maximisation of oil recovery from an oil-water separator sludge: Influence of type, concentration, and application ratio of surfactants', *Waste Management,* 82, pp. 100-110.

Ramirez, D., Kowalczyk, R. M. and Collins, C. D. (2019) 'Characterisation of oil sludges from different sources before treatment: High-field nuclear magnetic resonance (NMR) in the determination of oil and water content', *Journal of Petroleum Science and Engineering,* 174, pp. 729-737.

Tadros, T. F. (2005) 'Introduction', *Applied Surfactants: Principles and Applications*. Weinheim, Germany: Wiley-VCH Verlag GmbH and Co. KGaA, pp. 1-17.

Torres, L., Moctezuma, A., Avendaño, J. R., Muñoz, A. and Gracida, J. (2011) 'Comparison of bio- and synthetic surfactants for EOR', *Journal of Petroleum Science and Engineering,* 76(1–2), pp. 6-11.
